# Editorial: Insights in aging, metabolism and redox biology: 2024

**DOI:** 10.3389/fragi.2025.1750125

**Published:** 2025-12-09

**Authors:** Ruben K. Dagda, Jianhua Zhang

**Affiliations:** 1 Department of Pharmacology, University of Nevada, Reno School of Medicine Reno, Reno, NV, United States; 2 Department of Pathology, University of Alabama-Birmingham, Birmingham, AL, United States

**Keywords:** rapamycin, neurodegeneration, ROCK inhibitor, NAD+, lactate, autophagy and mitophagy, mitochondria and mitochondrial DNA, lifestyles including sleep/fasting/exercise

Roark and Iffland provide a comprehensive review of rapamycin’s emerging role as a potential off-label therapeutic for age-related diseases, including Alzheimer’s disease, as well as its proposed use in slowing the aging process. The authors examine how this natural immunosuppressant, which is best known for inhibiting the mTOR pathway, has been adopted by researchers, clinicians, and longevity enthusiasts as a putative “geroprotective” compound. The review synthesizes extensive preclinical evidence demonstrating that rapamycin reliably extends lifespan (>10%) across multiple model organisms, including *C. elegans*, *Drosophila*, and mice. At the same time, the authors underscore the significant risks associated with chronic rapamycin exposure in humans. Adverse effects such as recurrent infections and hyperlipidemia were first documented during clinical use of rapamycin analogs (e.g., sirolimus, everolimus) and later echoed anecdotally by self-experimenting “biohackers” exploring rapamycin as a promising anti-aging intervention. Finally, the review addresses important bioethical considerations surrounding rapamycin’s off-label use. The authors emphasize that aging is not formally classified as a disease and warn that unequal access to emerging longevity therapies risks deepening existing health disparities.

Mir et al. present a comprehensive review of the bidirectional relationships among sleep, redox metabolism, mitochondrial function, and brain bioenergetics, emphasizing their collective importance for brain health and systemic aging. The authors carefully outline the biology of sleep and detail how adequate sleep preserves neural function by (1) reducing oxidative stress through the upregulation of antioxidant defenses, (2) promoting the clearance of protein aggregates via autophagy, and (3) resetting key post-translational pathways that regulate mitochondrial dynamics, mitophagy, oxidative phosphorylation, calcium signaling, neuron–astrocyte metabolic coupling and circadian rhythms. Extending beyond the central nervous system, the review examines how sleep contributes to proper skeletal muscle maintenance, glucose homeostasis, gut integrity, microbial diversity, and the prevention of cellular senescence. Finally, the authors discuss lifestyle and pharmacological strategies, such as exercise, certain off-label therapeutics, melatonin, and flavonoids, which may enhance sleep quality by reducing oxidative stress, promoting autophagy, and modulating mTOR signaling. Importantly, they also caution that excessive antioxidant supplementation may paradoxically induce “antioxidant stress” and impair brain function.

Miller et al. provide a thorough and forward-looking review of preclinical models used to investigate mitochondrial diseases arising from mutations in the mitochondrial genome (mtDNA). The authors first provide a clear historical framework describing how cell-based systems and animal models harboring patient-derived mtDNA mutations, including those associated with LHON and MELAS, have advanced our understanding of disease mechanisms across tissues and organ systems. The review offers a well-balanced evaluation of key experimental platforms, including cybrid cell lines and three major mtDNA mouse models: xenomitochondrial mice, conplastic strains, and mitochondrial nuclear exchange (MNX) mice. Hence, by employing these models, researchers have uncovered important insights into how mtDNA haplogroups influence susceptibility to metabolic dysfunction, cardiomyopathy, and other chronic conditions. Finally, the authors highlight rapid technological progress that is reshaping the field. Advances in gene-editing tools (CRISPR–Cas9, mitoTALENs), induced pluripotent stem cell technologies, and organ-on-chip systems are enabling deeper mechanistic discovery and accelerating therapeutic development. Together, these innovations are paving the way for next-generation mitochondrial medicines aimed at treating monogenic mtDNA disorders in humans.

Zheng et al. provide a detailed and compelling review of Ras homolog–associated kinases (ROCK1 and ROCK2) as emerging therapeutic targets for Alzheimer’s disease (AD). The authors first contextualize the field’s longstanding emphasis on amyloid-β (Aβ 1-40/1-42) and tau, underscoring how downstream signaling pathways, in particular how the pathological role on the aberrant activation and overexpression of ROCK isoforms in the Alzheimer’s disease (AD) brain, has been comparatively underexplored despite their central roles in synaptic degeneration, mitochondrial dysfunction, oxidative stress, disrupted bioenergetics, and neuronal apoptosis *in vitro* and *in vivo* models of AD. A major strength of the review is its discussion of the complex and often compensatory interplay between ROCK1 and ROCK2; the authors highlight evidence showing that suppression of one isoform can unexpectedly increase expression or activity of the other in mouse models. These compensatory dynamics, along with the developmental consequences of global ROCK1/2 deletion, can complicate interpretation of preclinical findings. Such challenges have driven the development of brain-specific ROCK1 and ROCK2 knockout models, which the authors emphasize as powerful tools for resolving isoform-specific contributions to AD pathology. The review further summarizes extensive preclinical evidence demonstrating that selective inhibition of ROCK1, ROCK2, or dual ROCK inhibition produces distinct metabolic outcomes, including differential effects on oxidative phosphorylation, glycolysis, and overall mitochondrial physiology, while consistently lowering Aβ burden, reducing tau phosphorylation, restoring synaptic architecture, activating PINK1-Parkin–mediated mitophagy, and improving cognition in AD mouse models, including recent work from the authors’ own laboratory. Finally, the authors survey the broader clinical use of ROCK inhibitors in other chronic conditions highlighting their translational promise for AD while noting the pleiotropic side effects associated with non-selective ROCK inhibition.

Huo et al. investigated effects of overexpression of bacterial nicotinamidase PncA in mice on aging phenotypes in liver, kidney, heart and brain. NAD+ decreases with aging, and PncA is involved in intestinal microbiota and in converting NAM to NA which is an intermediate in one of the three key pathways of NAD+ biosynthesis. Female C57/BL6J mice were used in this study. In 8 months, old mice, PncA overexpression by AAV infection via tail vein resulted in increased NAD+ levels in the liver (also increased mtDNA/nuclear DNA ratio) and kidney but decreased NAD+ in the heart and hippocampus at 2 months post injection time point. In 4 months, old young mice, PncA overexpression accelerates the senescence of cardiac cells, decreased NAD+ levels, increased aging markers and cognitive decline. In contrast, in 25 months old mice, PncA overexpression increased SIRT1, and decreased p16, p53 and lipid droplets in the liver, consistent with delayed senescence. In the kidney, overexpression of PncA in the 25 months old mice increased Tfam, ATP5G1, and decreased IL6, with levels of these genes more resemble those in 4 months old mice. Although with caveats, including that only female mice were used, this study demonstrates that there is a clear tissue specific effect of the NAM to NA conversion on NAD+ and age-related phenotypes.

Ebrahimirad et al. reviewed 33 studies published between 2008 and 2023 on impacts of synthetic and natural compounds which have antioxidant-like cellular effects on oxidative stress and senescence markers. *In vivo*, *in vitro* and *ex vivo* studies were discussed. Aging related measurements include levels of reactive oxygen species, DNA damage, lysosomal activity, cytokine release, mitochondrial function, telomerase activity, stem cell markers and extracellular matrix integrity. NAC, TEMPOL, and metformin were compared to natural antioxidant TSG, curcumin, and ascorbic acid. Of note, this review also commented on challenges including the diverse doses used and variability of experimental models.

Lu et al. performed retrospective study on 156 Parkinson’s disease patients to determine the relationship between preoperative arterial blood lactate level and postoperative delirium (POD) after deep brain stimulation surgery. It is important to investigate mechanisms of POD as it is associated with prolonged hospitalization, cognitive decline and increased healthcare costs. Lactate levels have been previously shown to reflect tissue hypoperfusion, systemic inflammation, and are associated with multiple organ failure in trauma patients. Thus, this study tested if preoperative blood lactate levels are related to POD. From the 156 Parkinson’s disease patients who had underwent elective deep brain stimulation, 29 patients experienced POD during the three postoperative days. Using preoperative blood lactate levels as a continuous variable or as a binary variable, both indicate that high lactate is associated with higher risk of POD. Although the mechanisms of high blood lactate associates with postoperative POD are still unclear, and the sample size is still small, this study is of interest to researchers and physicians to further understand POD after deep brain stimulation in Parkinson’s disease patients.

Almuraikhy et al. compared metabolic signatures in serum samples in 29 randomized young non-obese healthy female subjects, between combined effects of 4 weeks exercise and Ramadan fasting and exercise alone. Data on 1039 known and 259 unknown identities were batch normalized. Major findings include many ceramides were lower in the combined exercise and fasting group, and discussed the implications.

We hope that this Research Topic provides new insights in aging research, in particular metabolism, redox regulation, protein quality control, and current development and challenges.

